# A genome-wide identification and expression analysis of the class III peroxidase gene family in *Mangifera indica* under abiotic stresses and the *MiPRX27* gene regulates oxidative stress

**DOI:** 10.1080/15592324.2025.2568933

**Published:** 2025-10-15

**Authors:** Jia Ran, Junliu Chen, Wang Hao, Hou Jiaxin, Min Zhu, Yeyuan Chen, Jinji Pu, He Zhang

**Affiliations:** a State Key Laboratory of Green Pesticide, Center for R&D of Fine Chemicals of Guizhou University, Guiyang, China; b National Key Laboratory for Tropical Crop Breeding, Key Laboratory of Integrated Pest Management on Tropical Crops, Ministry of Agriculture and Rural Affairs, Chinese Academy of Tropical Agricultural Sciences Environment and Plant Protection Institute, Haikou, China; c Tropical Crops Genetic Resources Institute, Chinese Academy of Tropical Agricultural Sciences, Haikou, China; d Sanya Research Institute of Chinese Academy of Tropical Agricultural Sciences, Sanya, China

**Keywords:** *MiPRX* genes, chromosomal localization, phylogenetic analysis, gene expression profiling, transgenic *Arabidopsis*, stress tolerance

## Abstract

Class III peroxidases (PRXs) are plant-specific enzymes that play vital roles in various physiological processes. However, the functional roles of mango *PRXs* under stress conditions remain poorly understood. In this study, we identified 76 *MiPRX* genes, which are unevenly distributed across the mango chromosomes. RT-qPCR analysis revealed differential expression of most *MiPRX* genes under oxidative, drought, and salt stress conditions, with *MiPRX27* showing a particularly prominent role. Under oxidative stress, heterologous overexpression of *MiPRX27* in *Arabidopsis* enhanced lateral root formation and accelerated root growth, suggesting that *MiPRX27* contributes to reducing plant sensitivity to oxidative stress. Overall, this study provides a theoretical foundation for further exploration of *MiPRX*-mediated mechanisms underlying mango stress tolerance.

## Introduction

Peroxidases (EC 1.11.1.X) are enzymes widely distributed across plants, animals, and microorganisms, where they catalyze the oxidation of various substrates using hydrogen peroxide as an electron acceptor.^1^ Based on the presence or absence of a heme prosthetic group, peroxidases are classified into heme and non-heme types.^2^ Heme peroxidases are further divided into two major groups: animal and non-animal peroxidases.^3^ In non-animal peroxidases, the heme group typically consists of protoporphyrin IX coordinated with iron (III). These enzymes are classified into three classes: class I, class II, and class III.^3^ Among these, Class III peroxidases (also referred to as PRX, PER, POD, or POX) are plant-specific secreted enzymes that play crucial roles in plant growth, development, signal transduction, metabolic regulation, and responses to both biotic and abiotic stresses.^2^^,^^4^^,^^5^

The functions of class III peroxidases in plants have been progressively elucidated. This gene family has been extensively studied in several species, including *Arabidopsis*,^6^ rice,^7^ tobacco,^8^ guava,^9^ cucumber,^10^ cotton,^11^ and others. Studies have shown that *TaPRX-2A* and *GsPOD40* enhance tolerance to salt and drought stresses by increasing the activity of antioxidant enzymes, such as superoxide dismutase (SOD), peroxidase (PRX), and catalase (CAT), while reducing reactive oxygen species (ROS)-induced oxidative damage.^12^^,^^13^ Under salt stress, *CRPRX1* was found to improve seed germination in tobacco.^14^ In response to arsenic (As) exposure, rice *OsPRX38* enhances resistance by upregulating the activities of SOD, PRX, and glutathione S-transferase (GST).^15^ At low temperatures, *Arabidopsis AtPRX62* and *AtPRX69* promote root hair development.^16^ *PRX* genes are also involved in hormone regulation, influencing processes such as seed germination,^17^ resistance to powdery mildew,^18^ and other disease-related processes. *PRX* genes have been extensively associated with enhanced resistance to various plant pathogens.^19^^,^^20^

Mango (*Mangifera indica* L.) is an evergreen woody plant belonging to the Anacardiaceae family, and it stands as one of the most economically significant fruit trees in tropical and subtropical regions. It originated in South Asia and has been cultivated for over 4000 y.^21^ The fruit is renowned for its high nutritional value,^22^ with mangiferin in particular exhibiting anti-cancer and anti-diabetic properties.^23-25^ However, various environmental factors, such as drought and salinity, significantly hinder the growth and development of mango.^26^^,^^27^ Salt and drought stress can lead to poor vegetative growth, reduced flowering, and increased fruit drop.^28^ Therefore, understanding the molecular mechanisms underlying mango response to abiotic stresses is crucial for improving its resilience.

To date, limited research has been conducted on the biological functions of the *PRX* gene family in mango. In this study, we identified the members of the *PRX* gene family in the mango genome through bioinformatics analysis. Subsequently, we analyzed gene structures, conserved protein motifs, chromosomal localization, biochemical properties, and phylogenetic relationships. The expression patterns of mango *PRX* genes under abiotic stresses, including salt, drought, and hydrogen peroxide, were examined using RT-qPCR. Notably, the biological function of *MiPRX27* in regulating oxidative stress was further investigated. This study lays the groundwork for further investigation into the roles of *PRX* genes in mango in response to abiotic stresses.

## Materials and methods

### Plant material, stress treatment

One-year-old mango plants (*Mangifera indica* L., cultivar 'Guifei') were used as experimental material. The plants were cultivated in a seedling nursery at the Key Laboratory of Integrated Pest Management for Tropical Crops in Haikou, Hainan Province, China, under controlled growth conditions: a temperature of 28 °C, a light intensity of 5,000 lux, 85% relative humidity, and a 16-hour light/8-hour dark photoperiod.^29^ Leaves were collected from healthy plants with uniform growth conditions at 0, 6, 12, 24, 48, and 72 h after root irrigation with oxidative stress (300 mmol·L⁻¹ H₂O₂), drought stress (30% PEG6000), or salt stress (300 mmol·L⁻¹ NaCl). Three biological replicates were used for each treatment.^28^^,^^30^^,^^31^ All samples were immediately frozen in liquid nitrogen and stored at −80 °C.

### Identification, annotation, and multiple sequence alignment of *MiPRX* genes

To identify *MiPRX* gene family members, genomic data for *Mangifera indica* (CATAS_Mindica_2.1) were retrieved from the NCBI Genome database (https://www.ncbi.nlm.nih.gov/datasets/genome/GCF_011075055.1/). Genomic data for *Arabidopsis thaliana* and *Oryza sativa* were obtained from the TAIR10 database (https://www.arabidopsis.org/) and the Ensembl Plants databases (https://plants.ensembl.org/index.html), respectively.

A total of 138 PRX protein sequences from *Oryza sativa* and 73 from *Arabidopsis thaliana* were used as reference sequences.^6^^,^^7^ Homology-based searches were performed across the entire mango genome using BLAST. Additionally, the conserved peroxidase domain (PF00141) was utilized to construct a Hidden Markov Model (HMM) for identifying candidate *PRX* genes within the mango genome. The Conserved Domain Database (CDD; https://www.ncbi.nlm.nih.gov/Structure/cdd/wrpsb.cgi) was used to validate the presence of the *PRX* domain. Candidate genes identified by both BLAST and HMM searches were intersected to cross-check and remove redundant sequences, thereby confirming the members of the *MiPRX* gene family. Redundant sequences were removed after comparison, and the remaining non-redundant sequences were considered potential MiPRX proteins. The physicochemical properties of the MiPRX proteins, including molecular weight (MW) and isoelectric point (pI), were predicted using the ProtParam tool (https://web.expasy.org/protparam/). Subcellular localization was predicted using Plant-mPLoc (http://www.csbio.sjtu.edu.cn/bioinf/plant-multi/).^32^ A phylogenetic tree was constructed using MEGA 11 (https://www.megasoftware.net/) with the neighbor-joining method and 1000 bootstrap replicates.^33^

The amino acid sequences of the *MiPRX* gene family were aligned using ClustalW (https://www.genome.jp/tools-bin/clustalw), based on the identified *MiPRX* genes. The alignment results were then visualized with ESPript 3 (https://espript.ibcp.fr/ESPript/ESPript/index.php) for further analysis.

### Analysis of conserved motifs, gene structure, and cis-acting elements of the *MiPRX* promoter

The conserved motifs of the *MiPRXs* were identified using the MEME online platform (https://meme-suite.org/meme/tools/meme),^34^ with a maximum of 10 motifs specified. The domain architecture of *MiPRXs* was further analyzed using the Batch CD-Search tool in the Conserved Domain Database (CDD) (https://www.ncbi.nlm.nih.gov/Structure/bwrpsb/bwrpsb.cgi), applying an E-value threshold of 0.01 and a maximum hit limit of 500. The chromosomal locations of *MiPRX* genes were determined using the GFF annotation file of the mango genome, and TBtools was used to visualize gene structures and conserved motifs.^35^ To explore the cis-acting elements in the promoter region of *MiPRX* genes, a 2000 bp sequence upstream of each *MiPRX* coding sequence (CDS) was extracted from the mango genome and defined as the promoter region. These sequences were submitted to the PlantCARE database (https://bioinformatics.psb.ugent.be/webtools/plantcare/html/) for cis-element prediction.^36^ The number, type, and distribution of the predicted elements were visualized using TBtools.^35^

### Chromosomal localization and duplication pattern analyzes

The chromosomal locations of *MiPRX* genes were obtained from the annotated mango genome. Gene duplication events were identified and visualized using TBtools, which was also employed to assign names to the *MiPRXs* based on their chromosomal positions.^35^ To explore the evolutionary relationships of Class III *PRXs* among *M. indica*, *A. thaliana*, and *O. sativa*, MCScanX was used.^35^

### RNA extraction and RT-qPCR

Total RNA was extracted using the RNA prep Pure Polysaccharide Polyphenol Plant Total RNA Extraction Kit (TIANGEN, Beijing, China), according to the manufacturer's instructions. First-strand cDNA synthesis was performed using the FastKing gDNA Dispelling RT SuperMix (TIANGEN, Beijing, China). Quantitative real-time PCR (RT-qPCR) was conducted with the SGExcel UltraSYBR Mixture (Sangon Biotech, Shanghai, China). Gene-specific primers were designed based on the coding sequences (CDS) of *MiPRXs* using Primer3Plus (https://www.primer3plus.com/), with *MiActin* used as the reference gene (Table S1). Gene expression was quantified using a qTOWER³ real-time PCR system (Analytik Jena GmbH, Göttingen, Germany).

### Construction of the expression vector and genetic transformation of *Arabidopsis*

The full-length coding region of *MiPRX27* was amplified by PCR using Pfu DNA polymerase and gene-specific primers (Table S1), then cloned into the pEGAD overexpression vector via homologous recombination. The resulting recombinant plasmid was designated 35S::*MiPRX27*. Both the recombinant and empty control vectors were introduced into *Agrobacterium tumefaciens* strain GV3101 using the heat shock method.^37^

### Transgenic *Arabidopsis* and stress treatment

*Arabidopsis* ecotype Col−0 was used as the wild-type control. Overexpression lines were generated via the floral dip method using *Agrobacterium tumefaciens* GV3101 harboring the 35S:*MiPRX27* recombinant plasmid.^38^ Transgenic plants were selected on medium containing 20 mg/L glufosinate ammonium and further confirmed by PCR. Sterilized seeds of both transgenic and Col−0 plants were sown on 1/2MS medium supplemented with oxidative stress (0.5 mmol·L⁻¹ H₂O₂), drought stress (200 mmol·L⁻¹ mannitol), or salt stress (150 mmol·L⁻¹ NaCl), and grown for 7 d.^39^ Primary root length, number of lateral roots, and total lateral root length were measured using ImageJ software to assess the tolerance of transgenic plants to oxidative, drought, and salt stresses.^40-42^ All experiments were performed with three independent biological replicates.

### Statistical analysis

All data were analyzed using a Student's t-test in IBM SPSS Statistics 26. *P*-values less than 0.05 were considered statistically significant and are represented by ‘*’. The relative mRNA levels for each gene were calculated using the 2^−ΔΔCT^ method.^29^ RT-qPCR experiments were performed at least three times under identical conditions. Results were visualized using GraphPad Prism 9 and TBtools.^29^^,^^35^ The experiment was conducted using three independent biological replicates.

## Results

### Genome-wide identification, phylogenetic analysis of MiPRX proteins

**Figure 1.**
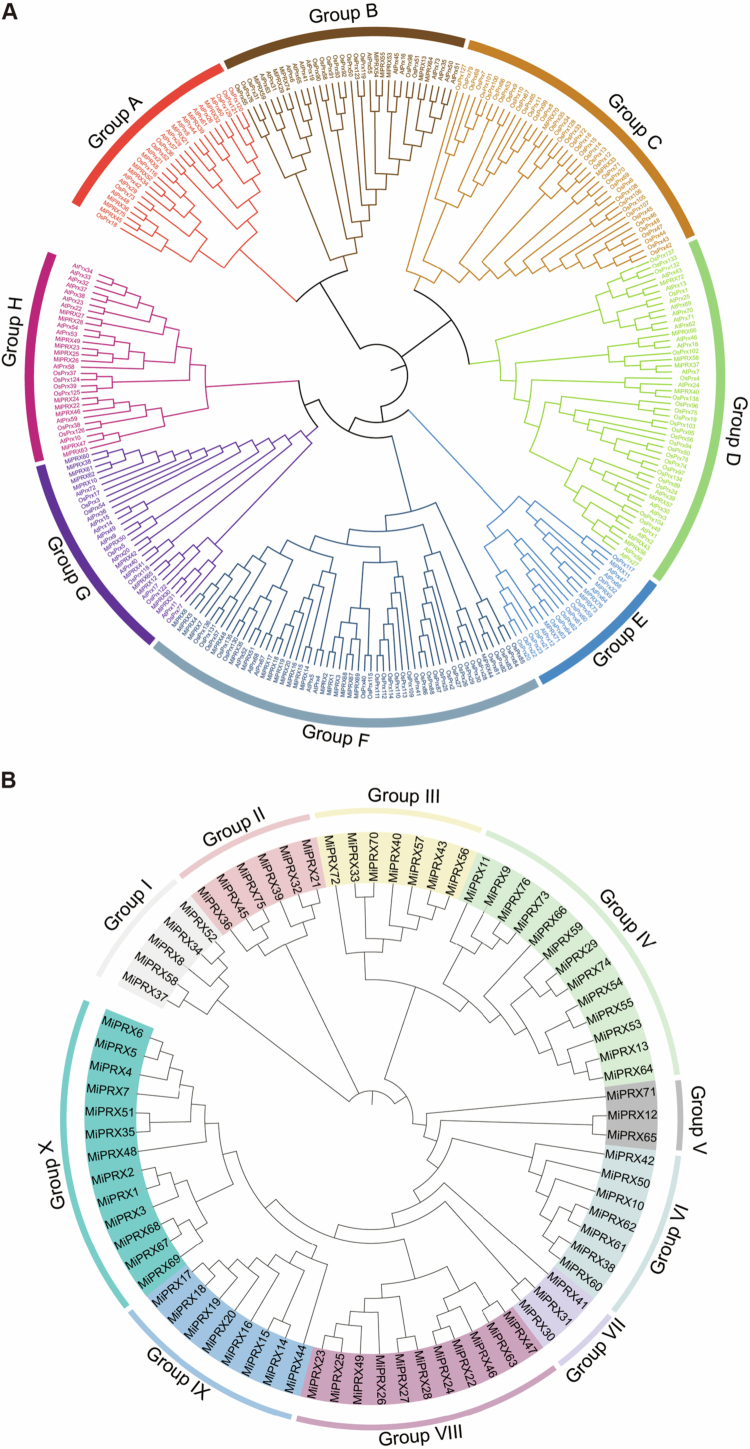
*MiPRXs* phylogenetic analysis. (A) Phylogenetic tree of PRX proteins from *O. sativa*, *A. thaliana*, and *M. indica*. Peroxidases from *M. indica*, *A. thaliana*, and *O. sativa* are denoted as *MiPRX*, *AtPRX*, and *OsPRX*, respectively. (B) Phylogenetic analysis of mango *PRXs*. Different colors represent different groups.

Using both BLAST and HMM methods, we identified 76 MiPRX proteins from the mango genome. Based on their chromosomal locations, these *MiPRX* family members were named *MiPRX1* to *MiPRX76* (Table S2). The MiPRX proteins varied significantly in length, ranging from 216 amino acids (*MiPRX38*) to 500 amino acids (*MiPRX36*), with molecular weights (Mw) ranging from 24.01 to 53.56 kDa. Additionally, the isoelectric points (pI) of the MiPRX proteins varied between 4.25 (*MiPRX23*) and 9.64 (*MiPRX38*). Predicted subcellular localization indicated that MiPRX proteins were primarily localized in the cytoplasm, with some also detected in vesicles (Table S2). A phylogenetic tree was constructed using the neighbor-joining (NJ) method to analyze the evolutionary relationships of *PRX* amino acid sequences from *M. indica*, *A. thaliana*, and *O. sativa*. The tree also facilitated the comparison of monocotyledonous and dicotyledonous plants. Eight Groups (A-H) were identified in the phylogenetic tree, as shown in Figure 1A. Group F contained the largest number of MiPRX proteins (21 members), followed by Group G (12 members). In contrast, Group C had only two members, representing 4.76% of the total in this group. To further investigate the evolutionary relationships among *MiPRX* members, a separate phylogenetic tree based solely on *MiPRX* amino acid sequences was also constructed (Figure 1B). Multiple sequence alignments of 76 *MiPRX* sequences were performed using ClustalW and ESPript 3 (Figure S1). Ten groups (Group I–X) were defined based on the 76 *MiPRX* members in mango. Notably, the distribution of *MiPRX* proteins across the groups varied, with group sizes ranging from 3 to 13 members per group. Group IV and Group X contained the highest number of members, with 13 in each, while Group V contained only three members, accounting for 3.9% of the total.

### Analysis of conserved motifs and gene structure of *MiPRXs*

**Figure 2.**
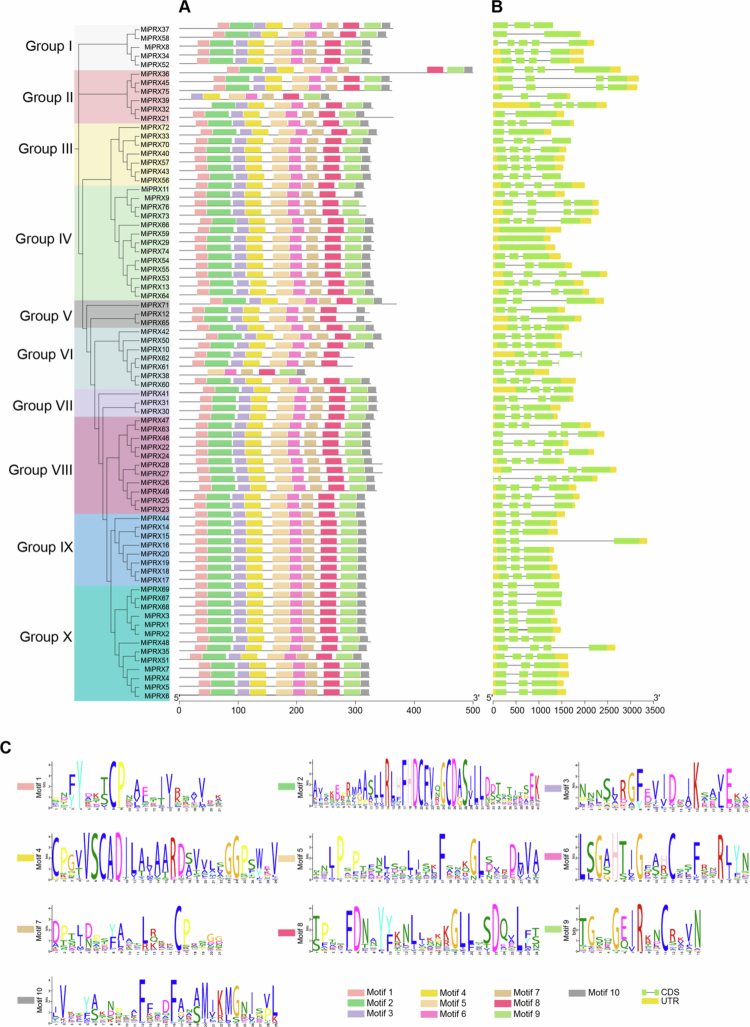
Conserved protein motifs and gene structure of *MiPRXs*. (A) Conserved motifs of *MiPRXs*. Different colored boxes represent conserved motifs, while gray lines indicate non-conserved motifs. (B) Exon-intron structures of the *MiPRX* genes. Green squares represent exons, yellow squares indicate untranslated regions (UTRs), and gray lines represent introns. (C) The amino acid sequences of the motifs. Larger letters indicate a higher frequency of amino acids at that position.

In this study, we explored the evolutionary conservation of *MiPRX* genes by analyzing their structural characteristics. A total of at least ten motifs were identified in the amino acid sequences of *MiPRXs* using the MEME database, as shown in Figure 2. Based on these motifs, *MiPRXs* were clustered into groups in the evolutionary tree, suggesting that these proteins may share functional similarities (Figure 2A). At least six conserved motifs are present in all MiPRX proteins. For example, motifs 5, 6, 7, 8, 9, and 10 are found in *MiPRX38*. Interestingly, motifs 5, 6, 7, and 8 are conserved across all MiPRX proteins. The amino acid composition of these conserved motifs is highly similar. Over 86.84% of all MiPRX proteins from mango contain all ten motifs.

We also performed exon-intron analysis to explore the structural diversity of *MiPRX* genes (Figure 2B). The results revealed that *MiPRX* genes contain between 0 and 5 introns. Notably, *MiPRX8*, *MiPRX61*, and *MiPRX62* each contained four introns, while *MiPRX29*, *MiPRX59*, and *MiPRX74* lack introns. All members of Group IX possess two introns and three exons. These findings indicate that genes within the same group tend to exhibit similar structural features.

The conserved motifs of *MiPRXs* were identified using the MEME tool, and the specific amino acid sequences of 10 conserved motifs were obtained (Figure 2C, Table S3). The cis-acting elements of *MiPRXs* were identified using PlantCARE and visualized using TBtools. Cis-acting elements can be divided into eight categories based on their function (Figure S2).

### Chromosomal distribution of the *MiPRX* genes

**Figure 3.**
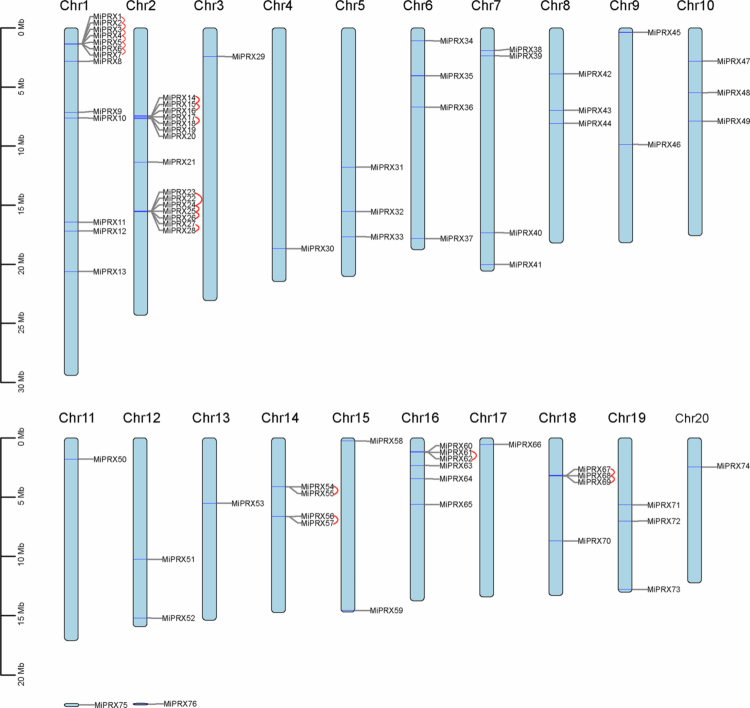
Chromosomal distribution and tandem duplications of *MiPRXs*. The *MiPRX* genes, from *MiPRX1* to *MiPRX76*, were renamed based on their chromosomal positions, with two genes remaining unassigned to any chromosome. Chromosomal localization of *MiPRXs* was carried out using the mango genome database. Tandem duplications of two pairs are indicated by red lines.

Statistics on the distribution of the mango *PRX* gene family showed that 76 *MiPRX* genes are distributed across the chromosomes of mango. Each chromosome contains between 1 and 15 *MiPRX* genes, with approximately one-third of these genes located on Chr1 and Chr2 (Figure 3). On Chr3, Chr 4, Chr 11, Chr 13, and Chr 20, only one *MiPRX* gene is present. Additionally, *MiPRX75* and *MiPRX76* are located in unassembled chromosomal regions.

### Syntenic and selection pressure analysis of the *MiPRX* gene family

**Figure 4.**
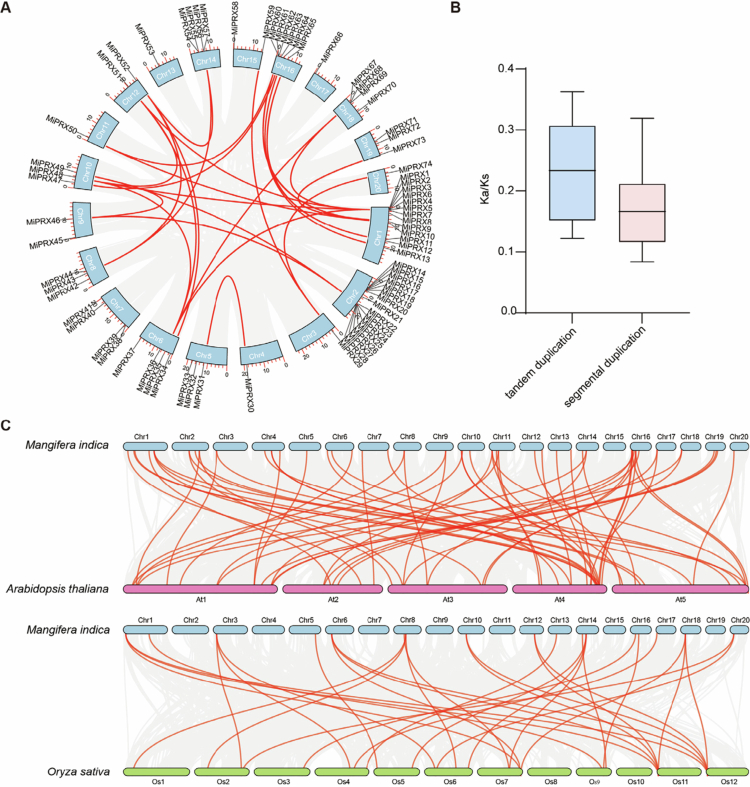
Syntenic relationship o*f MiPRX* genes. (A) *MiPRX* genes duplication analysis. Gray lines represent duplicated gene pairs in mango, while red lines highlight the segmental duplication events of *MiPRX* genes. (B) Distribution of Ka/Ks values for *MiPRXs* duplication events. (C) *PRX* genes synteny analysis between *O. sativa*, *A. thaliana*, and *M. indica*. Chromosomes of *M. indica*, *A. thaliana*, and *O. sativa* are distinguished by different colors. Syntenic *PRX* gene pairs are highlighted by red lines, and collinear blocks are represented by gray lines.

Gene family expansion is primarily driven by gene duplication. To investigate the expansion mechanism of *MiPRXs*, we performed collinearity analysis (Figure 4, Table S4). Among the 43 paralogous pairs of *MiPRXs*, 18 pairs exhibited tandem duplication events, while 25 pairs showed segmental duplication (Figures 3 and 4A). These results indicate that both tandem and segmental duplications contribute to the expansion of *MiPRXs*. Additionally, the Ka/Ks ratio for both tandem and segmental duplications is less than 1, suggesting negative selection (Figure 4B, Table S4). This implies that the evolution of class III peroxidases is largely influenced by purifying selection, with tandem duplications playing a more significant role.

To better understand the evolutionary relationships among *PRX* genes in plants, we analyzed the collinearity between *M. indica* and *A. thaliana*, as well as between *M. indica* and *O. sativa* (Figure 4C). A total of 63 homologous gene pairs were identified between *M. indica* and *A. thaliana*, and 32 pairs between *M. indica* and *O. sativa* (Figure 4C, Table S5). In these gene pairs, a single *MiPRX* may correspond to multiple genes in *A. thaliana* and *O. sativa*. For example, *MiPRX29* was found to homologous to *AT1G24110.1*, *AT5G40150.1*, *Os02t0741200-01*, *Os04t0465100-01*, and *Os06t0237600-00*. These collinearity results suggest that the evolutionary relationship between *M. indica* and *A. thaliana* is closer than that between *M. indica* and *O. sativa.*

### *MiPRX* gene expression patterns under oxidative, drought, and salt treatments

**Figure 5.**
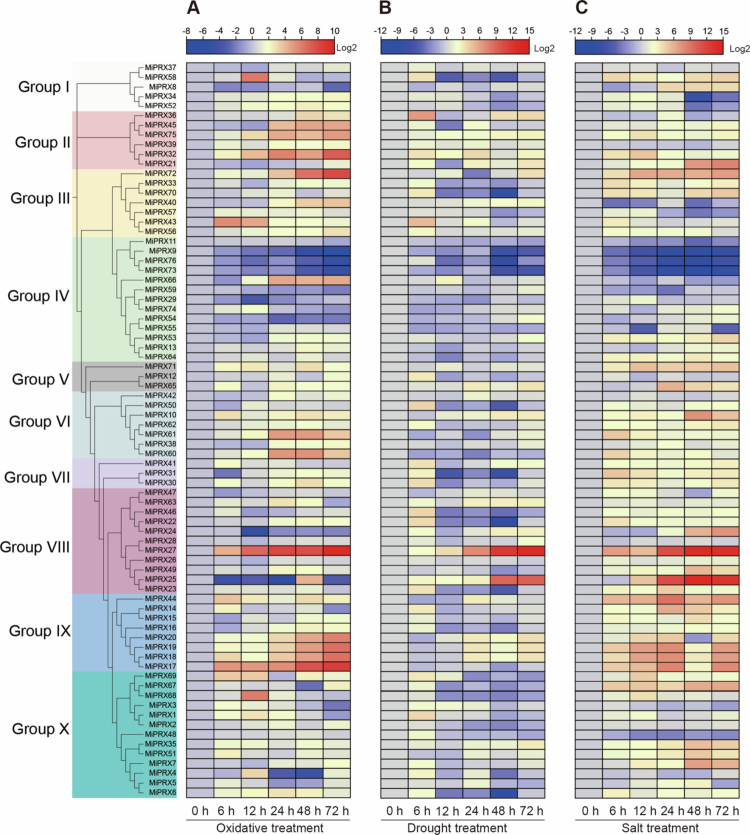
The expression patterns of *MiPRXs* under various stress conditions were analyzed using RT-qPCR. We evaluated the *MiPRXs* expression profiles in response to oxidative treatment (A), drought treatment (B), and salt treatment (C). Fold changes were calculated using Log 2-based RT-qPCR, and a heat map was generated using TBtools. Relative expression was considered down-regulated if it was less than 0.5-fold of the control and up-regulated if it was 1.5-fold higher than the control. Different colors visually represent the relative expression levels.

To further elucidate the potential biological functions of *MiPRXs* under abiotic stresses, we analyzed their expression patterns in response to oxidative, drought, and salt stresses using RT-qPCR (Figure 5). The results show that the expression levels of *MiPRXs* vary under different stress conditions, indicating their diverse roles in stress responses. Expression of 76 *MiPRXs* was evaluated under oxidative stress (Figure 5A, Table S6). Notably, *MiPRX6,* −*10*, −*17*, −*18*, −*19*, −*20*, −*27*, −*32*, −*34*, −*35*, −*39*, −*43*, −*44*, −*52*, −*56*, −*57*, −*60*, −*61*, −*71*, −*72*, and −*75* exhibited high expression levels at various time points. In contrast, *MiPRX7,* −*22*, −*31*, −​​​​​​​*36*, −​​​​​​​*38*, −​​​​​​​*40*, −​​​​​​​*42*, −​​​​​​​*45*, ​−​​​​​​*36*, and −​​​​​​​*53* showed increased expression after 12 h (Figure 5A). Specifically, *MiPRX4* and *MiPRX68* reached their peak expression at 12 h, while *MiPRX25* and *MiPRX43* exhibited the highest expression at 6 and 48 h, respectively. Additionally, several *MiPRXs*, including *MiPRX9*, *MiPRX73*, and *MiPRX76*, were down-regulated under stress conditions.

Following drought stress treatment, 13.1% of *MiPRX* members exhibited increased expression levels (Figure 5B, Table S7), with *MiPRX25* and *MiPRX27* showing the highest expression. In contrast, *MiPRX9*, −​​​​​​​*11*, −​​​​​​​*73*, and −​​​​​​​*76* were down-regulated under drought conditions. Notably, except for *MiPRX66*, which showed induced expression at 12 h, all genes in Group IV exhibited decreased expression at one or more time points. Additionally, the expression of *MiPRX18* and *MiPRX19* peaked at 6 h and then gradually reduced. *MiPRX9*, −​​​​​​​*11*, −​​​​​​​*73*, and −​​​​​​​*76* showed reduced transcript levels, indicating their sensitivity to drought stress.

The expression levels of Group IV members (*MiPRX9*, −​​​​​​​*11*, −​​​​​​​*59*, −​​​​​​​*66*, −​​​​​​​*73*, −​​​​​​​*76*) were down-regulated at most time points following salt stress treatment (Figure 5 C, Table S8). *MiPRX25*, −​​​​​​​*27*, and −​​​​​​​*72* were rapidly activated and maintained at high expression levels throughout the treatment. In contrast, *MiPRX17*, −​​​​​​​*18*, and −​​​​​​​*19* exhibited a pattern of initial increase followed by a decrease, peaking at 36 h and reaching their lowest levels at 48 h.

**Figure 6.**
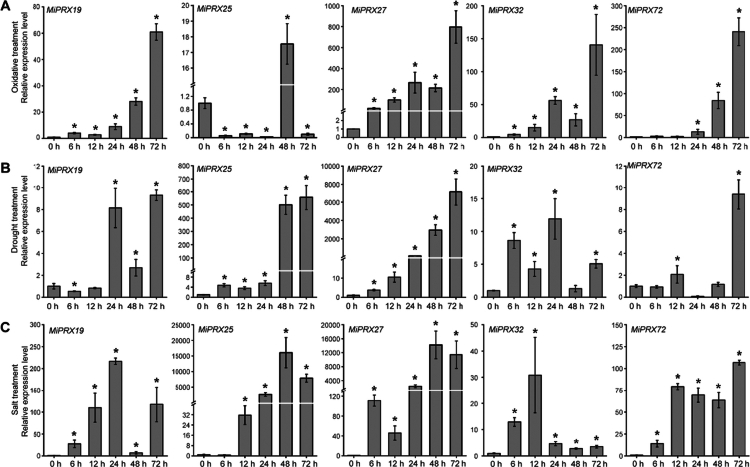
The relative expression levels of *MiPRX19*, −​​​​​​​25, −​​​​​​​27, −​​​​​​​32, and −​​​​​​​72 in response to oxidative, drought, and salt treatments were assessed using RT-qPCR. Figures (A-C) show the expression levels under oxidative (A), drought (B), and salt treatment (C). The 2^−∆∆CT^ method was used to calculate expression levels (*; *p*-value ≤ 0.05). Three biological and technical replicates were used to calculate the mean and standard deviation (SD).

The RT-qPCR analysis of *MiPRXs* under abiotic stresses revealed that *MiPRX19*, −​​​​​​​*25*, −​​​​​​​*27*, −​​​​​​​*32*, and −​​​​​​​*72* respond positively to oxidative, drought, and salt treatments. Under oxidative stress, *MiPRX27* expression levels increased over time (Figure 6A). Conversely, the expression levels of *MiPRX25* and *MiPRX27* increased following exposure to drought and salt stresses (Figure 6B–C). Under all three abiotic stress conditions, the expression levels of *MiPRX27* consistently increased over time. These results suggest that *MiPRX27* may play a significant role in the stress resistance mechanisms of mango.

### Heterologous overexpression of *MiPRX27* affects sensitivity to oxidative, drought, and salt stress in *Arabidopsis thaliana*

To further validate the role of *MiPRX27* in mango resistance to abiotic stress, we stably overexpressed the *MiPRX27* gene in *Arabidopsis thaliana* using Agrobacterium-mediated stable genetic transformation. Transgenic lines were screened using Sanger sequencing to identify a *MiPRX27*-OE4 line without nucleotide mutations. To assess the function of *MiPRX27* in abiotic stress, we measured the primary root length, lateral root number, and total lateral root length of Col−0 and *MiPRX27*-OE overexpression lines under oxidative, drought, and salt stress treatments, respectively.

**Figure 7.**
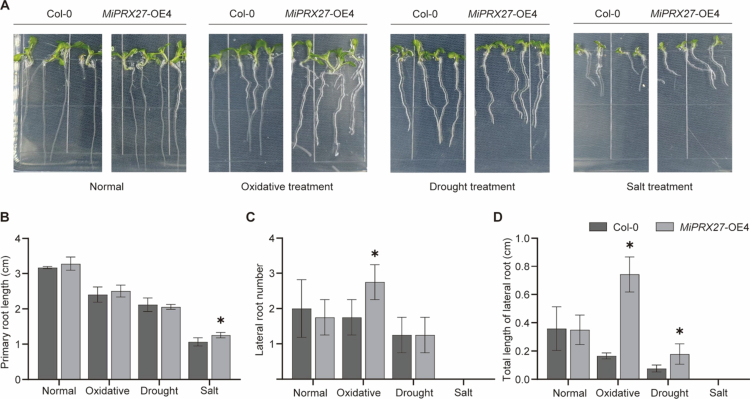
Growth of 7-d-old *Arabidopsis* seedlings in Col−0, *MiPRX27* overexpression lines was observed under different stresses in 1/2 MS medium supplemented with oxidative, drought, and salt treatments. Phenotypes of *Arabidopsis* seedlings of the Col−0 and *MiPRX27*-OE4 overexpression lines under different stresses (A), primary root length (B), lateral root number (C), Total length of lateral root (D) (*; *p*-value ≤ 0.05, Student's t-test).

As shown in Figure 7, under normal 1/2MS culture conditions, there were no significant differences in primary root length, lateral root number, or total lateral root length between the two lines (Figure 7A). However, under oxidative stress, the lateral root number and total root length of *MiPRX27*-OE were significantly greater than those of Col-0 (Figure 7C). Under drought treatment, *MiPRX27*-OE exhibited a greater total lateral root length compared to Col-0 (Figure 7D). In response to salt treatment, the primary root length of *MiPRX27*-OE was significantly increased relative to Col-0 (Figure 7B).

## Discussion

Class III peroxidases (PRXs) play a crucial role in regulating various physiological processes, including plant growth and development. They play a key role in responding to both biotic and abiotic stresses by controlling reactive oxygen species (ROS) and other oxidative molecules.^1^^,^^3^ Under abiotic stress, PRX enzymes can scavenge reactive oxygen species (ROS), thereby mitigating associated damage.^42^ To date, the *PRX* gene family has been extensively studied at the genome-wide level in *Arabidopsis*, rice, tobacco,^8^ pineapple,^43^ and cassava.^5^ However, limited research has been conducted on the *PRX* gene family in mango. Following the completion of the mango genome sequencing, the *PRX* gene family has been analyzed at the genomic level.^44^ In this study, we identified 76 *MiPRX* genes using mango genomic data, and analyzed their phylogenetic tree, collinearity, gene structures, conserved motifs, and expression patterns. This study lays the foundation for understanding the role of *MiPRXs* in abiotic stresses.

This study identified 76 *MiPRX* proteins, each containing 10 highly conserved motifs. In addition, variations in the number and type of conserved motifs were observed across the 76 MiPRX proteins. The diversity of these motifs may be implicated in the biological functions of *MiPRXs*. Variations in gene structures are critical for the evolution of gene families.^45^ Gene structural analysis revealed that the 76 *MiPRX* genes differ in the number of exons and introns, with clear structural similarities observed within each phylogenetic group. Additionally, some *MiPRX* genes lack introns, likely due to specific evolutionary mechanisms such as exon or intron gain or loss.^46^

The *MiPRX* members are unevenly distributed on 20 chromosomes, a pattern consistent with the distribution of *PRX* genes on chromosomes in cassava,^5^ birch, castor bean,^47^ sugarcane,^48^ and ginger.^45^ Gene family expansion is typically driven by segmental duplications, tandem duplications, and genome-wide duplications.^49^ Analysis of *MiPRXs* duplication events revealed that 58.1% of the duplicated genes resulted from segmental duplications, suggesting that segmental duplications are the primary mechanism driving *MiPRXs* evolution. In contrast, *ShPRXs* in sugarcane are predominantly expanded through tandem duplications. Ka/Ks analysis further indicates that the evolution of the *MiPRX* family is largely shaped by negative selection, a pattern consistent with findings in soybean,^13^ tobacco,^8^ and rice.^7^ Homology analysis of *PRX* genes revealed that mango shares a higher number of homologous genes and exhibits closer genetic relationships with *Arabidopsis thaliana*.

Previous studies have demonstrated that peroxidase (PRXs) genes play a crucial role in regulating both abiotic stresses and environmental stress resistance. These studies have examined the differential expression patterns of *PRXs* in response to salt, drought, heavy metals, oxidative stress, in species such as cucumber,^10^ sugarcane,^48^ and *Arabidopsis*.^50^ Under oxidative stress, transgenic soybean *GsPRX9* exhibited enhanced antioxidant capacity, primarily by increasing the activities of PRX, SOD, and GST enzymes and reducing intracellular H_2_O_2_ levels.^51^ Under salt stress, *PRX* scavenges ROS to alleviate the detrimental effects of salinity. In tobacco, *NtPRX63* upregulates the expression of antioxidant-related genes, enhancing antioxidant enzyme activity and ROS scavenging ability, thereby improving salt stress tolerance.^52^ Similarly, salt stress significantly induced the activity of the sweet potato *IbPRX17*, reducing intracellular ROS levels and mitigating salt-induced damage.^53^ During drought stress, the *ZmPRX26* gene in maize plays a crucial role. The expression patterns of *MiPRXs* in response to H_2_O_2_, drought, and salt treatments showed differential transcriptional regulation in response to these stresses. Notably, the transcriptional level of *MiPRX27* was significantly activated, indicating that *MiPRX27* may play a key role in stress responses.

To investigate the response of *MiPRX27* to abiotic stress, we introduced *MiPRX27* into *Arabidopsis thaliana*. Seeds were cultured under oxidative stress (0.5 mmol·L⁻¹ H₂O₂), drought stress (200 mmol·L⁻¹ mannitol), and salt stress (150 mmol·L⁻¹ NaCl). Under normal 1/2 MS medium, no significant differences in taproot and lateral root growth were observed between wild-type and *MiPRX27* overexpressing lines. However, under oxidative stress, the number and length of lateral roots in the overexpressing lines were significantly greater than those in the wild-type. These results suggest that *MiPRX27* overexpression reduces the sensitivity of *Arabidopsis* lateral roots to oxidative stress (Figure 7), highlighting the specific role of *MiPRX27* in regulating plant responses to oxidative stress.

## Conclusion

This study provides a comprehensive genome-wide analysis of the *MiPRX* gene family in mango, identifying a total of 76 *MiPRX* members. Phylogenetic and structural analyzes revealed conserved motifs and key evolutionary patterns within the family. Additionally, *MiPRX27* was found to be upregulated under oxidative, drought, and salt stress conditions, suggesting its potential role in enhancing tolerance to abiotic stresses. Overexpression of *MiPRX27* in *Arabidopsis* was achieved using Agrobacterium-mediated floral dip transformation, and the transgenic lines were assessed for stress resistance under various conditions. Notably, under oxidative stress, *MiPRX27* overexpression significantly increased the number and length of lateral roots in *Arabidopsis*. This study provides valuable insights into the functional roles of *PRX* genes and offers a rich genetic resource for developing mango varieties with improved stress resistance.

## Supplementary Material

Supplementary material

## Data Availability

The data that support the findings of this study are available in the Supporting Information for this article. Supplementary figures and tables related to the study can be found in the Supporting Materials, Figure S1-S2, Table S1-S8.

## References

[1] Kidwai M, Ahmad IZ, Chakrabarty D. Class III peroxidase: an indispensable enzyme for biotic/abiotic stress tolerance and a potent candidate for crop improvement. Plant Cell Rep. 2020;39:1381–1393. doi: 10.1007/s00299-020-02588-y. 32886139

[2] Hiraga S, Sasaki K, Ito H, Ohashi Y, Matsui H. A large family of class III plant peroxidases. Plant Cell Physiol. 2001;42:462–468. doi: 10.1093/pcp/pce061. 11382811

[3] Freitas CDT, Costa JH, Germano TA, Rocha RD, Ramos M, Bezerra LP. Class III plant peroxidases: from classification to physiological functions. Int J Biiol Macromol. 2024;263:130306. doi: 10.1016/j.ijbiomac.2024.130306. 38387641

[4] Almagro L, Ros LVG, Belchi-Navarro S, Bru R, Barceló AR, Pedreño MA. Class III peroxidases in plant defence reactions. J Exp Bot. 2009;60:377–390. doi: 10.1093/jxb/ern277. 19073963

[5] Wu CL, Ding XP, Ding ZH, Tie WW, Yan Y, Wang Y, Yang H, Hu W. The class III peroxidase (POD) gene family in *Cassava*: identification, phylogeny, duplication, and expression. Int J Mol Sci. 2019;20:2730. doi: 10.3390/ijms20112730. 31163686 PMC6600411

[6] Tognolli M, Penel C, Greppin H, Simon P. Analysis and expression of the class III peroxidase large gene family in *Arabidopsis thaliana*. Gene. 2002;288:129–138. doi: 10.1016/S0378-1119(02)00465-1. 12034502

[7] Passardi F, Longet D, Penel C, Dunand C. The class III peroxidase multigenic in land plants family in rice and its evolution. Phytochem. 2004;65:1879–1893. doi: 10.1016/j.phytochem.2004.06.023. 15279994

[8] Cheng LT, Ma LX, Meng LJ, Shang HH, Cao PJ, Jin JJ. Genome-wide identification and analysis of the class III peroxidase gene family in tobacco (*Nicotiana tabacum*). Front Genet. 2022;13:916867. 10.3389/fgene.2022.916867. 35769995 PMC9234461

[9] Gull S, Ali MM, Ejaz S, Ali S, Rasheed M, Yousef AF, Stępień P, Chen F. Comprehensive genomic exploration of class III peroxidase genes in guava unravels physiology, evolution, and postharvest storage responses. Sci Rep. 2024;14:1446. doi: 10.1038/s41598-024-51961-4. 38228714 PMC10791677

[10] Luo WR, Liu JJ, Xu WC, Zhi SS, Wang XD, Sun YD. Molecular characterization of peroxidase (PRX) gene family in *Cucumber*. Genes. 2024;15:1245. doi: 10.3390/genes15101245. 39457369 PMC11507654

[11] Yang LP, Xia YD, Fei CM, Shahzad K, Niu M, Feng JJ, Ma J, Wang X, Song J, Xu S, et al. Genome-wide analysis of Class III peroxidase (PRX) family core genes and functional mechanism of *GhPRXR1-A* for seed development in *Gossypium hirsutum*. Int J Biiol Macromol. 2025;295:139529. doi: 10.1016/j.ijbiomac.2025.139529. 39761879

[12] Su PS, Yan J, Li W, Wang L, Zhao JX, Ma X, Kong L. A member of wheat class III peroxidase gene family, *TaPRX-2A*, enhanced the tolerance of salt stress. BMC Plant Biol. 2020;20:392. doi: 10.1186/s12870-020-02602-1. 32847515 PMC7449071

[13] Aleem M, Riaz A, Raza Q, Aleem M, Aslam M, Kong KK, Atif RM, Kashif M, Bhat JA, Zhao T. Genome-wide characterization and functional analysis of class III peroxidase gene family in soybean reveal regulatory roles of *GsPOD40* in drought tolerance. Genomics. 2022;114:45–60. doi: 10.1016/j.ygeno.2021.11.016. 34813918

[14] Kumar S, Jaggi M, Sinha AK. Ectopic overexpression of vacuolar and apoplastic *Catharanthus roseus* peroxidases confers differential tolerance to salt and dehydration stress in transgenic tobacco. Protoplasma. 2012;249:423–432. doi: 10.1007/s00709-011-0294-1. 21643888

[15] Kidwai M, Dhar YV, Gautam N, Tiwari M, Ahmad IZ, Asif MH, Chakrabarty D. Oryza sativa class HI peroxidase (*OsPRX38*) overexpression in *Arabidopsis thaliana* reduces arsenic accumulation due to apoplastic lignification. J Hazard Mater. 2019;362:383–393. doi: 10.1016/j.jhazmat.2018.09.029. 30245406

[16] Pacheco JM, Ranocha P, Kasulin L, Fusari CM, Servi L, Aptekmann AA, Gabarain VB, Peralta JM, Borassi C, Marzol E, et al. Apoplastic class III peroxidases *PRX62* and *PRX69* promote *Arabidopsis* root hair growth at low temperature. Nat Commun. 2022;13:1310. doi: 10.1038/s41467-022-28833-4. 35288564 PMC8921275

[17] Gao W, Jiang YT, Yang XH, Li T, Zhang LT, Yan SN, Cao J, Lu J, Ma C, Chang C. Functional analysis of a wheat class III peroxidase gene, *TaPer12-3A*, in seed dormancy and germination. BMC Plant Biology. 2024;24:318. doi: 10.1186/s12870-024-05041-4. 38654190 PMC11040755

[18] Zhao YW, Li WK, Wang CK, Sun Q, Wang WY, Huang XY, Xiang Y, Hu D. MdPRX34L, a class III peroxidase gene, activates the immune response in apple to the fungal pathogen *Botryosphaeria dothidea*. Planta. 2024;259:86. doi: 10.1007/s00425-024-04355-9. 38453695

[19] Zheng C, Wang XM, Xu Y, Wang SM, Jiang X, Liu XL, Cui W, Wu Y, Yan C, Lu Y, et al. The peroxidase gene *OsPrx114* activated by *OsWRKY50* enhances drought tolerance through ROS scavenging in rice. Plant Physiol Biochem. 2023;204:108138. doi: 10.1016/j.plaphy.2023.108138. 39492168

[20] Li Q, Qin XJ, Qi JJ, Dou WF, Dunand C, Chen SC, He Y. CsPrx25, a class III peroxidase in *Citrus sinensis*, confers resistance to citrus bacterial canker through the maintenance of ROS homeostasis and cell wall lignification. Hortic Res. 2020;7:192. doi: 10.1038/s41438-020-00415-9. 33328465 PMC7705758

[21] Jagarlamudi S, Rosaiah G, Kurapati RK, Pinnamaneni R. Molecular identification of Mango, *Mangifera indica* L. var. totupura. Bioinformation. 2011;5:405–409. doi: 10.6026/97320630005405. 21423885 PMC3055156

[22] Lebaka VR, Wee YJ, Ye WB, Korivi M. Nutritional composition and bioactive compounds in three different parts of mango fruit. Int J Environ Res Public Health. 2021;18:741. doi: 10.3390/ijerph18020741. 33467139 PMC7830918

[23] Oliver-Simancas R, Muñoz R, Díaz-Maroto MC, Pérez-Coello MS, Alañón ME. Mango by-products as a natural source of valuable odor-active compounds. J Sci Food Agric. 2020;100:4688–4695. doi: 10.1002/jsfa.10524. 32418224

[24] Oliver-Simancas R, Díaz-Maroto MC, Pérez-Coello MS, Alañón ME. Viability of pre-treatment drying methods on mango peel by-products to preserve flavouring active compounds for its revalorisation. J Food Eng. 2020;279:109953. doi: 10.1016/j.jfoodeng.2020.109953.

[25] Qu SH, Wang WQ, Li DP, Li SM, Zhang LK, Fu YH, Zhang N. Mangiferin inhibits mastitis induced by LPS via suppressing *NF-κB* and *NLRP3* signaling pathways. Int Immunopharmacol. 2017;43:85–90. doi: 10.1016/j.intimp.2016.11.036. 27984712

[26] Hou XB, Kong Y, Teng Z, Yang CF, Li YF, Zhu ZJ. Integrating genes and metabolites: unraveling mango's drought resilience mechanisms. BMC Plant Biol. 2024;24:208. doi: 10.1186/s12870-024-04908-w. 38519933 PMC10960439

[27] Helaly MN, El-Hoseiny H, El-Sheery NI, Rastogi A, Kalaji HM. Regulation and physiological role of silicon in alleviating drought stress of mango. Plant Physiol Biochem. 2017;118:31–44. doi: 10.1016/j.plaphy.2017.05.021. 28603082

[28] Wang HL, Chen ZL, Luo RX, Lei C, Zhang MT, Gao AP, Pu J. Caffeic acid O-methyltransferase gene family in mango (*Mangifera indica* L.) with transcriptional analysis under biotic and abiotic stresses and the role of *MiCOMT1* in salt tolerance. Int J Mol Sci. 2024;25:2639. doi: 10.3390/ijms25052639. 38473886 PMC10931984

[29] Lei C, Dang Z, Zhu M, Zhang M, Wang H, Chen Y, Zhang H. Identification of the *ERF* gene family of *Mangifera indica* and the defense response of *MiERF4* to *Xanthomonas campestris* pv. mangiferaeindicae. Gene. 2024;912:148382. doi: 10.1016/j.gene.2024.148382. 38493974

[30] Liu ZL, Luo C, Dong L, Toan CV, Wei PX, He XH. Molecular characterization and expression analysis of a GTP-binding protein (*MiRab5*) in *Mangifera indica*. Gene. 2014;540:86–91. doi: 10.1016/j.gene.2014.02.022. 24560931

[31] Zhang H, Liu ZX, Luo RX, Sun Y, Yang CF, Li X, Gao A, Pu J. Genome-wide Characterization, identification and expression profile of *MYB* transcription factor gene family during abiotic and biotic stresses in mango (*Mangifera indica*). Plants-basel. 2022;11:3141. doi: 10.3390/plants11223141. 36432870 PMC9699602

[32] Chou KC, Shen HB. Plant-mPLoc: a top-down strategy to augment the power for predicting plant protein subcellular localization. PLoS One. 2010;5:e11335. doi: 10.1371/journal.pone.0011335. 20596258 PMC2893129

[33] Tamura K, Stecher G, Kumar S. MEGA11 molecular evolutionary genetics analysis version 11. Mol Biol Evol. 2021;38(7):3022–3027. doi: 10.1093/molbev/msab120. 33892491 PMC8233496

[34] Bailey TL, Boden M, Buske FA, Frith M, Grant CE, Clementi L, Ren J, Li WW, Noble WS. MEME SUITE: tools for motif discovery and searching. Nucleic Acids Res. 2009;37:W202–W208. doi: 10.1093/nar/gkp335. 19458158 PMC2703892

[35] Chen CJ, Wu Y, Li JW, Wang X, Zeng ZH, Xu J, Liu Y, Feng J, He Y, Xia R. TBtools-II: A “one for all, all for one” bioinformatics platform for biological big-data mining. Mol Plant. 2023;16:1733–1742. doi: 10.1016/j.molp.2023.09.010. 37740491

[36] Rombauts S, Déhais P, Van Montagu M, Rouzé P. PlantCARE, a plant cis-acting regulatory element database. Nucleic Acids Res. 1999;27:295–296. doi: 10.1093/nar/27.1.295. 9847207 PMC148162

[37] Xie Y, Tan HJ, Ma ZX, Huang JR. DELLA proteins promote anthocyanin biosynthesis via sequestering *MYBL2* and JAZ Suppressors of the *MYB/bHLH/WD40* Complex in *Arabidopsis thaliana*. Mol Plant. 2016;9:711–721. doi: 10.1016/j.molp.2016.01.014. 26854848

[38] Ali I, Sher H, Ali A, Hussain S, Ullah Z. Simplified floral dip transformation method of *Arabidopsis thaliana*. J Microbiol Meth. 2022;197:106492. doi: 10.1016/j.mimet.2022.106492. 35597520

[39] Jia T, Hou JR, Iqbal MZ, Zhang YZ, Cheng BZ, Feng HH, Li Z, Liu L, Zhou J, Nie G, et al. Overexpression of the white clover *TrSAMDC1* gene enhanced salt and drought resistance in *Arabidopsis thaliana*. Plant Physiol Biochem. 2021;165:147–160. doi: 10.1016/j.plaphy.2021.05.018. 34038811

[40] Chu YY, Duan RC, Song HR, Zhang WS, Zhou YX, Ma YT, Yin X, Tian L, Ausin I, Han Z. *AtHD2D* is involved in regulating lateral root development and participates in abiotic stress response in *Arabidopsis*. J Plant Physiol. 2024;297:154242. doi: 10.1016/j.jplph.2024.154242. 38614048

[41] Hernández-Esquivel AA, Torres-Olmos JA, Méndez-Gómez M, Castro-Mercado E, Flores-Cortéz I, Peña-Uribe CA, Campos-García J, López-Bucio J, Reyes-de la Cruz H, Valencia-Cantero E, et al. Hydrogen peroxide modulates the expression of the target of rapamycin (TOR) and cell division in *Arabidopsis thaliana*. Protoplasma. 2024;261:1147–1158. doi: 10.1007/s00709-024-01959-6. 38802622

[42] Yang JJ, Zhang GQ, An J, Li QX, Chen YH, Zhao XY, Wu J, Wang Y, Hao Q. Expansin gene *TaEXPA2* positively regulates drought tolerance in transgenic wheat (*Triticum aestivum* L.). Plant Sci. 2020;298:110596. doi: 10.1016/j.plantsci.2020.110596. 32771153

[43] Hou XW, Lu ZW, Hong KQ, Song KH, Gu H, Hu W, Yao Q. The class III peroxidase gene family is involved in ascorbic acid induced delay of internal browning in pineapple. Front Plant Sci. 2022;13 953623. doi: 10.3389/fpls.2022.953623. 35991401 PMC9382127

[44] Wang P, Luo YF, Huang JF, Gao SH, Zhu GP, Dang ZG, Gai J, Yang M, Zhang H, Ye X, et al. The genome evolution and domestication of tropical fruit mango. Genome Biol. 2020;21:60. doi: 10.1186/s13059-020-01959-8. 32143734 PMC7059373

[45] Gong M, Jiang YJ, Tang SH, Xing HT, Li H, Gu JJ, Mao M, Wang W, Xia M. Genome-wide identification of the class III peroxidase gene family in ginger and expression analysis under high temperature and intense light stress. Horticulturae. 2024;10:911. doi: 10.3390/horticulturae10090911.

[46] Xu GX, Guo CC, Shan HY, Kong HZ. Divergence of duplicate genes in exon-intron structure. Proc Natl Acad Sci U S A. 2012;109:1187–1192. doi: 10.1073/pnas.1109047109. 22232673 PMC3268293

[47] Li JY, Fan MB, Yang SQ, Huo HY, Fan WQ, Lue SY, Lü S, Zhang J. Genome-wide identification of castor bean class III peroxidase genes and analysis of expression patterns under abiotic stresses. BMC Plant Biol. 2025;25:900. doi: 10.1186/s12870-025-06945-5. 40646456 PMC12247346

[48] Shang HY, Fang LQ, Qin LF, Jiang HT, Duan ZZ, Zhang H, Yang Z, Cheng G, Bao Y, Xu J, et al. Genome-wide identification of the class III peroxidase gene family of sugarcane and its expression profiles under stresses. Front Plant Sci. 2023;14 1101665. doi: 10.3389/fpls.2023.1101665. 36794222 PMC9924293

[49] Panchy N, Lehti-Shiu M, Shiu SH. Evolution of gene duplication in plants. Plant Physiol. 2016;171:2294–2316. doi: 10.1104/pp.16.00523. 27288366 PMC4972278

[50] Eljebbawi A, Savelli B, Libourel C, Estevez JM, Dunand C. Class III peroxidases in response to multiple abiotic stresses in *Arabidopsis thaliana* Pyrenean Populations. Int J Mol Sci. 2022;23:3960. doi: 10.3390/ijms23073960. 35409333 PMC8999671

[51] Jin T, Sun YY, Zhao RR, Shan Z, Gai JY, Li Y. Overexpression of peroxidase gene *GsPRX9* confers salt tolerance in *Soybean*. Int J Mol Sci. 2019;20:3745. doi: 10.3390/ijms20153745. 31370221 PMC6695911

[52] Lu LM, Yang SY, Liu L, Lu YF, Yang SM, Liu F, Ni S, Zeng F, Ren B, Wang X, et al. Physiological and quantitative proteomic analysis of *NtPRX63*-overexpressing tobacco plants revealed that *NtPRX63* functions in plant salt resistance. Plant Physiol Biochem. 2020;154:30–42. doi: 10.1016/j.plaphy.2020.04.022. 32521442

[53] Zhang H, Wang Z, Li X, Gao XR, Dai ZR, Cui YF, Zhi Y, Liu Q, Zhai H, Zhao N, et al. The *IbBBX24-IbTOE3-IbPRX17* module enhances abiotic stress tolerance by scavenging reactive oxygen species in sweet potato. New Phytol. 2022;233:1133–1152. doi: 10.1111/nph.17860. 34773641

