# Supplementary material for: A genome-wide identification and expression analysis of the class III peroxidase gene family in Mangifera indica under abiotic stresses and the MiPRX27 gene regulates oxidative stress
Source: Plant Signal Behav. 2025 Oct 15;20(1):2568933. doi: 10.1080/15592324.2025.2568933 (PMC12530489; doi:10.1080/15592324.2025.2568933)
Supplement: Supplementary material [file KPSB_A_2568933_SM1486.zip › Figure S2.docx]

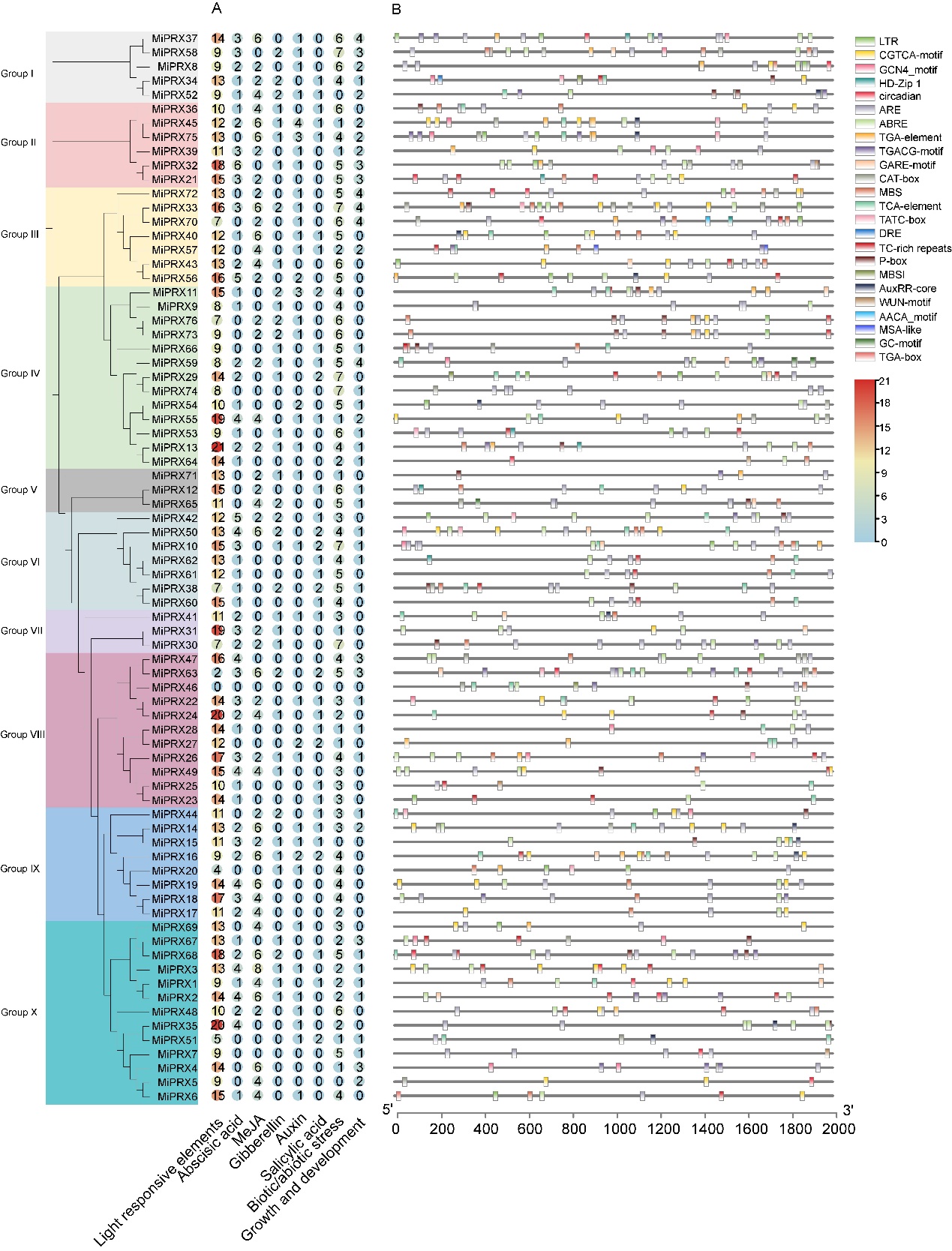


**Figure S2**. Prediction of cis-acting elements in *MiPR*X promoters. (A) The promoters of *MiPRXs* can be classified into light-responsive, ABA, MeJA, GA, IAA, SA, abiotic stress-related elements, growth, and development-responsive elements. (B) Visualisation and analysis of component types and locations using TBtools.
