# Supplementary figures and images for: A genome-wide identification and expression analysis of the class III peroxidase gene family in Mangifera indica under abiotic stresses and the MiPRX27 gene regulates oxidative stress
Source: Plant Signal Behav. 2025 Oct 15;20(1):2568933. doi: 10.1080/15592324.2025.2568933 (PMC12530489; doi:10.1080/15592324.2025.2568933)

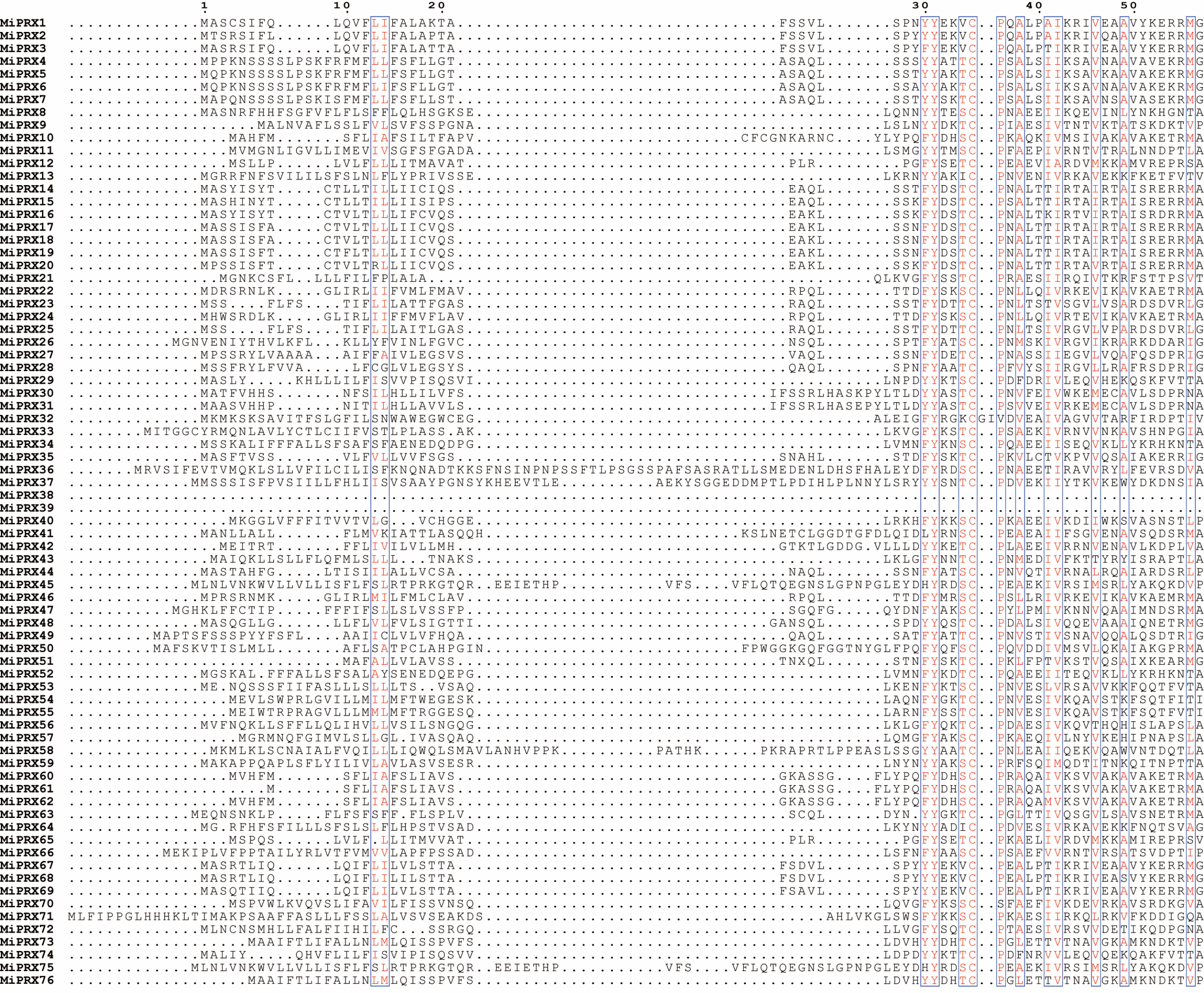


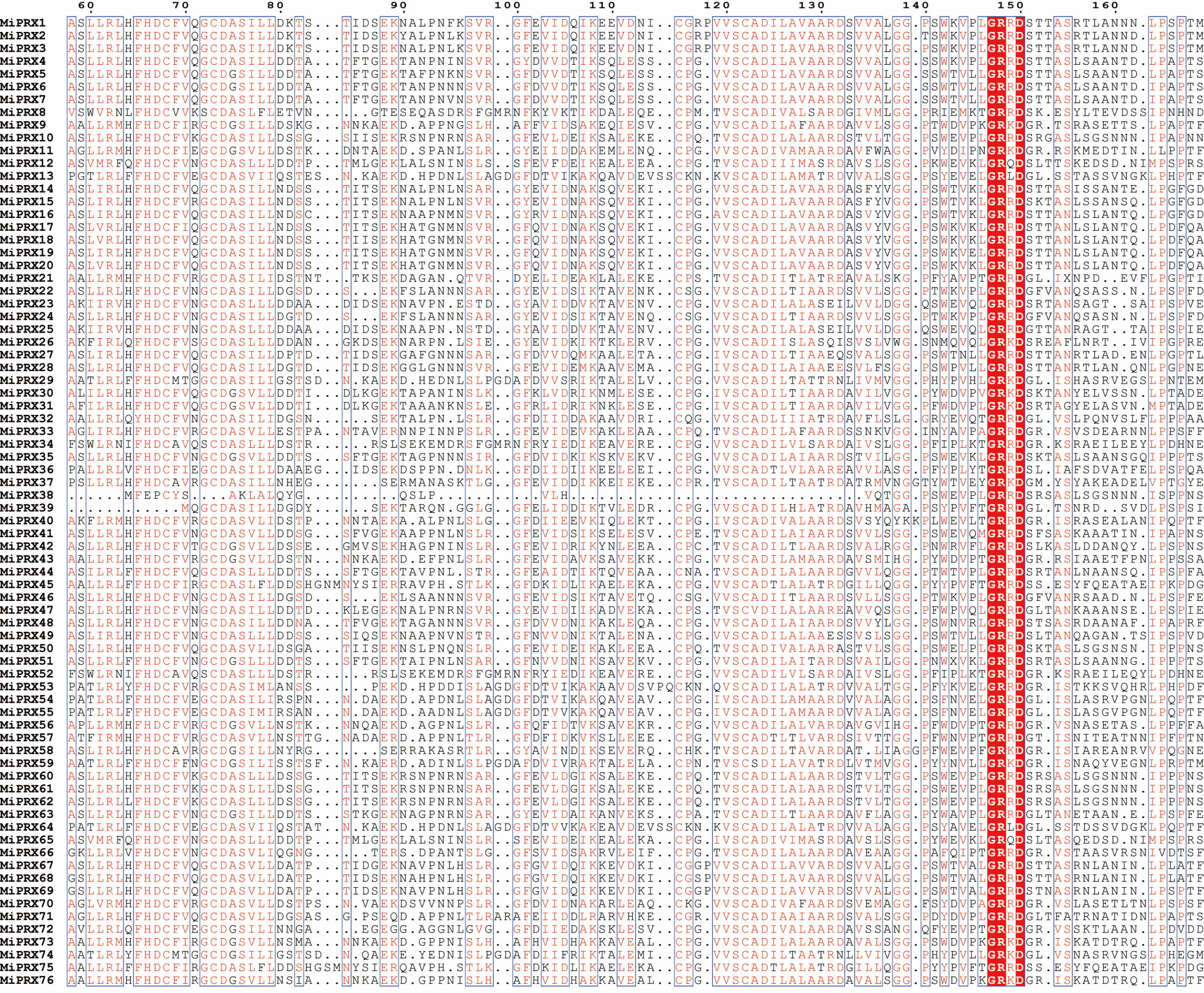


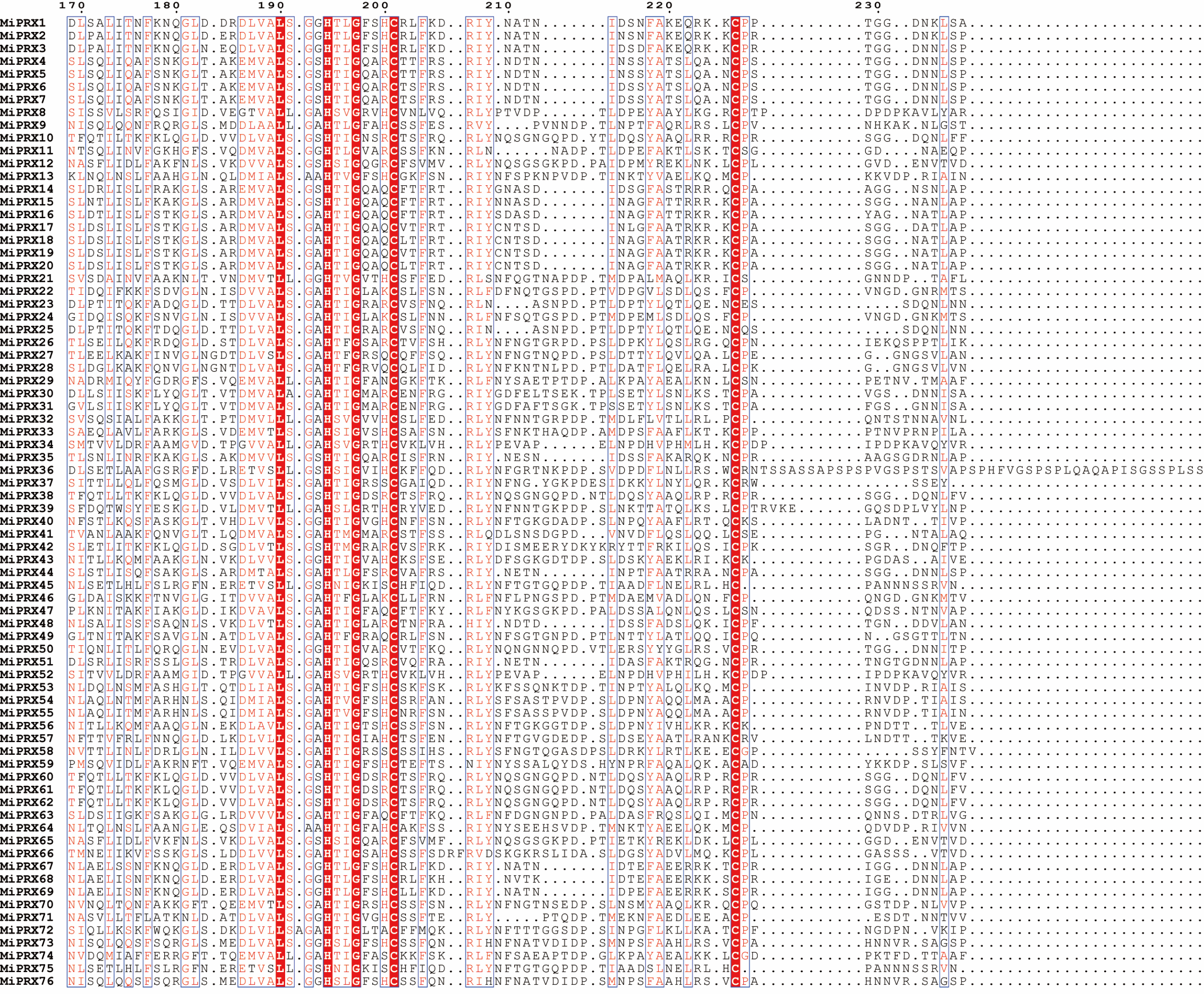


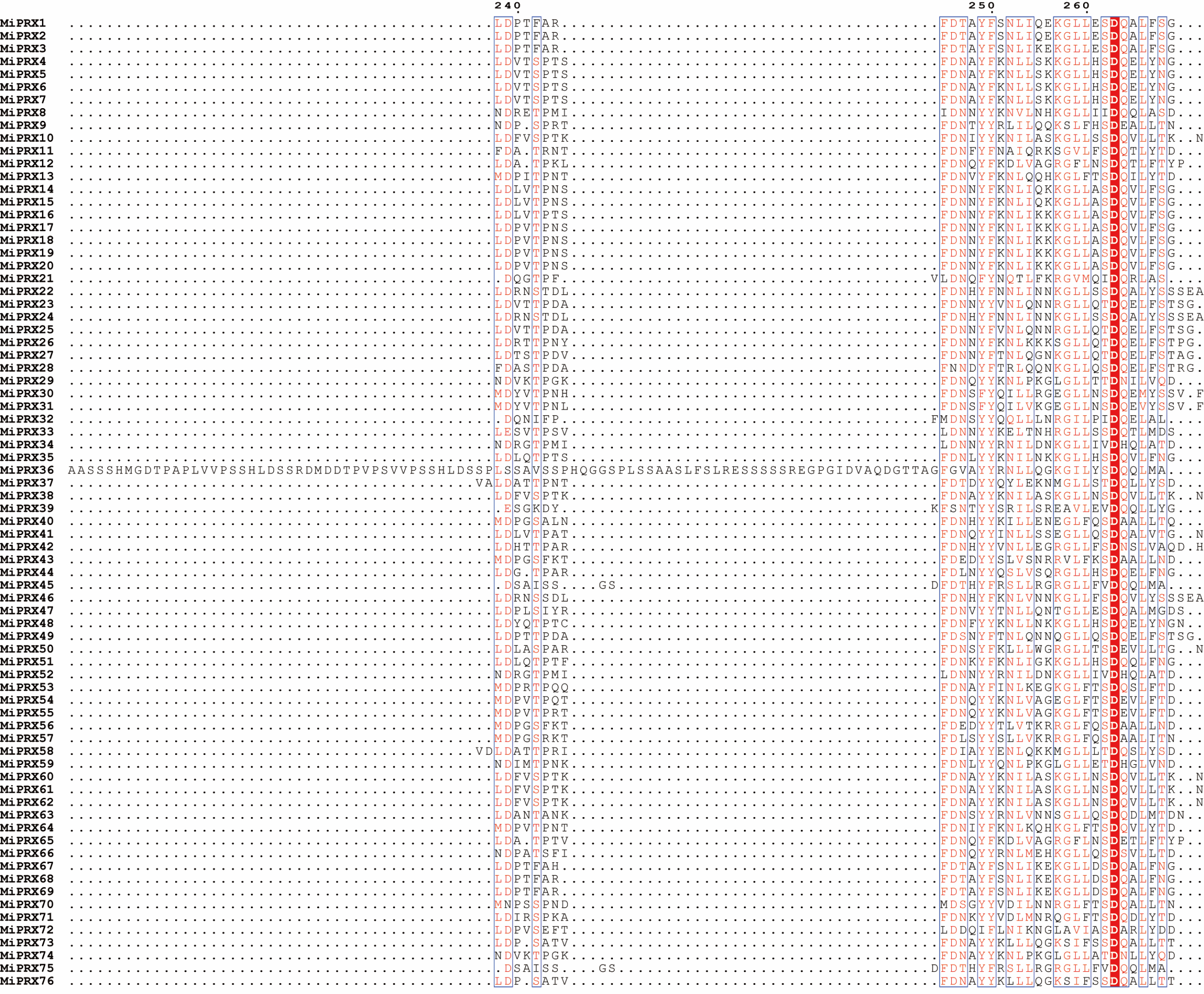


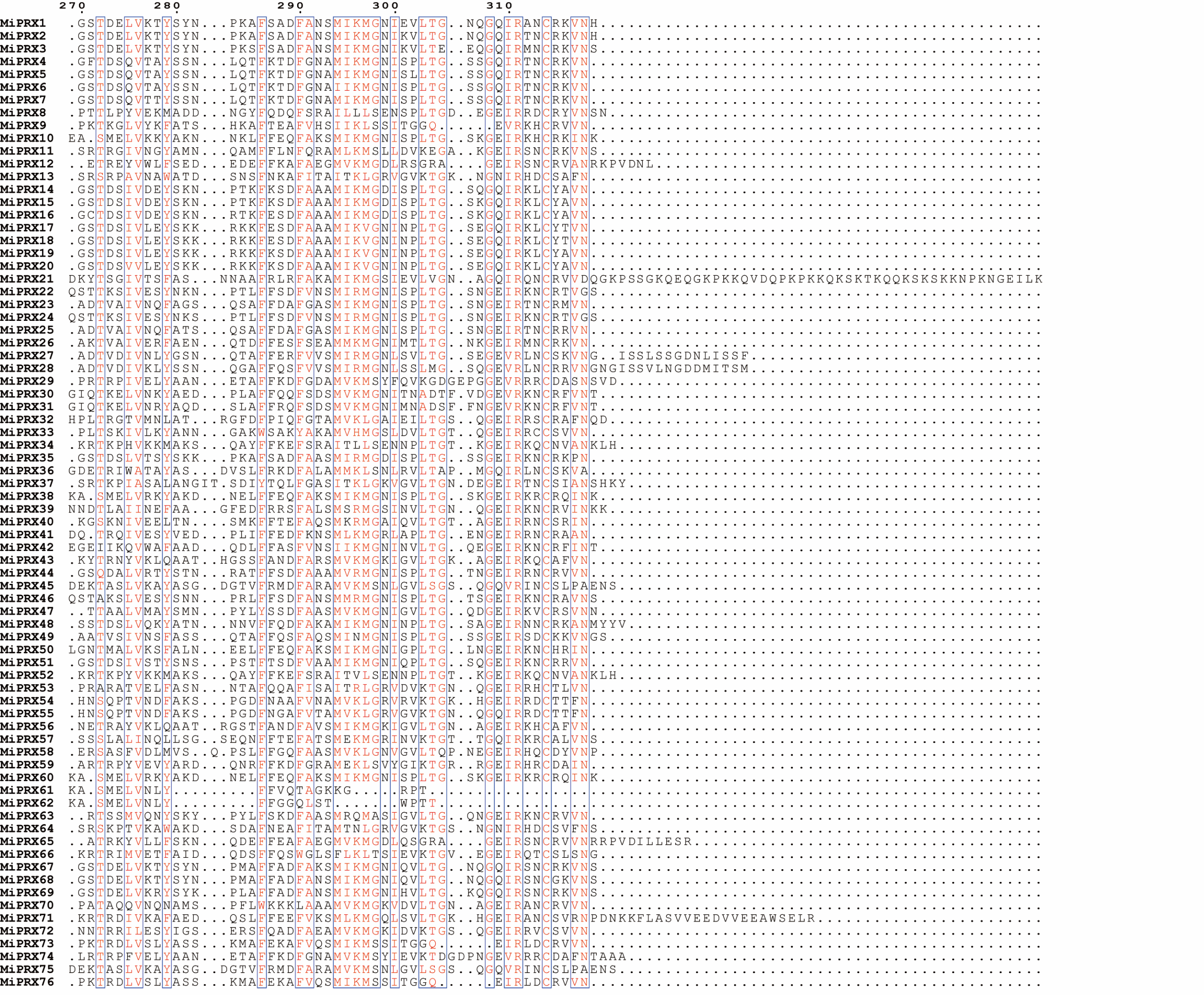


**Figure S1**. Multiple sequence alignment of *MiPRXs*.

Supplement: Supplementary material [file KPSB_A_2568933_SM1486.zip › Figure S1.docx]
